# Evaluating Ylehd, a recombinant epoxide hydrolase from *Yarrowia lipolytica* as a potential biocatalyst for the resolution of benzyl glycidyl ether†

**DOI:** 10.1039/c8ra00628h

**Published:** 2018-04-06

**Authors:** Chandrika Bendigiri, K. Harini, Sajal Yenkar, Smita Zinjarde, R. Sowdhamini, Ameeta RaviKumar

**Affiliations:** Institute of Bioinformatics and Biotechnology, Savitribai Phule Pune University Pune 411 007 India ameeta@unipune.ac.in; Department of Biotechnology, Savitribai Phule Pune University Pune 411 007 India; National Centre for Biological Sciences Bengaluru India

## Abstract

Glycidyl ethers and their vicinal diols are important building blocks in the organic synthesis of anti-cancer and anti-obesity drugs. Ylehd, an epoxide hydrolase from tropical marine yeast *Yarrowia lipolytica*, was explored for its enantioselective properties by kinetic, thermodynamic and *in silico* studies. Kinetic resolution of racemic phenyl glycidyl ether (PGE) yielded (*S*)-epoxide while for benzyl glycidyl ether (BGE) (*R*)-epoxide was obtained, with vicinal diols of the opposite configuration. Amongst the enantiomers of PGE and BGE, the (*S*)-selective conversion of benzyl glycidyl ether to its corresponding diol, (*S*)-3-benzyloxy-1,2-propanediol while retaining (*R*)-BGE was most favourable with 95% ee in 20 min. Enantioselective conversion of specific enantiomer of BGE to its corresponding diols was attributed to the favourable kinetic and thermodynamic parameters as well as to the number and proximity of water molecules near the base H325 in the active site pocket. The easily available and highly active Ylehd could be a potential biocatalyst for large scale preparation of pharmaceutically relevant chiral (*R*)-benzyl glycidyl ether and (*S*)-3-benzyloxy-1,2-propanediol.

## Abstract 2

Ylehd, an enantioselective epoxide hydrolase with potential application in resolution of racemic benzyl glycidyl ether.

## Graphical abstract

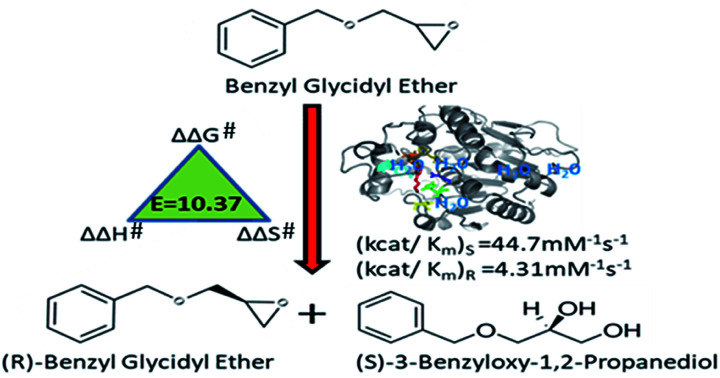

## Introduction

**Fig. 1:**
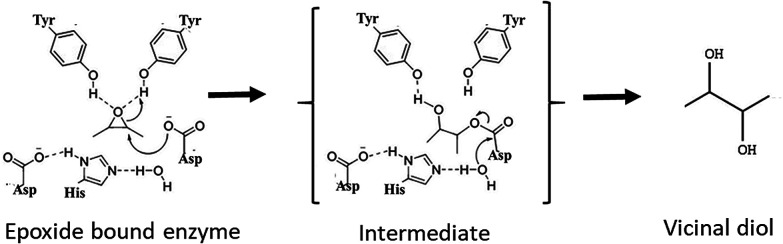
Generalized reaction mechanism for epoxide hydrolase.

Chiral epoxides and their corresponding vicinal diols are essential and valuable synthons for the preparation of enantiopure biologically active compounds. Chemical routes for the synthesis of these enantiopure epoxides and diols involve asymmetric epoxidation or dihydroxylation of olefin substrates carried out with catalysts such as chiral salen cobalt complexes and porphyrin manganese adducts.^1,2^ However, these reactions employ toxic chemical reagents and generate by-products that render the process non-ecofriendly. Hence, alternative simple and green biological routes using enantioselective enzymes such as epoxide hydrolase (EH) is a promising approach.^3^ EHs are stable, co-factor-independent and selectively hydrolyse one enantiomer of racemic epoxide into the corresponding vicinal diol by addition of a molecule of water, leaving the slow reacting enantiomer. Therefore, effective production of optically pure epoxides depends on the appropriate EH as well as the substrate building block. EHs (EC 3.2.2.3) belonging to the α/β hydrolase superfamily are ubiquitously found in nature.^4^ They catalyse the conversion of epoxides to their corresponding vicinal diols through a hydrolytic mechanism as shown in Fig. 1 involving a catalytic triad comprising of a nucleophile (Asp), a general base (His) and an acid residue (Asp/Glu) with positioning and activation of the substrate done by two tyrosines. As per the mechanism proposed for murine EH (PDB 1CQZ), catalysis involves a nucleophilic attack by an active site Asp leading to the formation of an acyl-enzyme intermediate complex. In the next step(s), a charge relay system comprising of the acidic residue (Asp/Glu) and base histidine, which abstracts a proton from the water molecule, thereby activating it and hydrolysing the acyl-enzyme intermediate resulting in the release of the diol product.^5^

In recent times, microbial EH enzymes, especially from fungi and yeasts have gained importance in resolution of racemic epoxides. Kinetic resolution of styrene oxide (SO) and its derivatives has been well studied using EH from *Aspergillus* sp., *Rhodotorula glutinis* and *Sphingomonas* sp. with high yields and enantiomeric excess (ee) of 98–99.5%.^6–8^ Asymmetric hydrolysis of aliphatic epoxides mediated by yeasts have also been reported.^9^ Another class of epoxides, the aryl glycidyl(oxiran-2-ylmethyl) ethers such as phenyl glycidyl ether (PGE) and benzyl glycidyl ethers (BGE) and their diols are useful intermediates for synthesis of chirally relevant compounds in pharmaceutical, agrochemical and fine chemical industries. Kinetic resolution of PGE and its derivatives has been well studied^10,11^ for the synthesis of β-blockers in cardiovascular therapies. For PGE, while bacterial EHs selectively yield (*S*)-epoxide (>99% ee) and (*R*)-diols (89% ee), the yeast cells of *Trichosporon loubierii* yielded (*R*)-epoxide with good enantioselectivity (99% ee) and (*S*)-diols (33% ee) depending on the cell/substrate ratio.^12^ In contrast, benzyl glycidyl ether (BGE) and its derivatives, important building blocks for anti-cancer and anti-obesity drugs, have been less explored. For example, (*R*)-BGE is used as a starting compound in the chemical synthesis of anti-obesity drug, (−)-tetrahydrolipstatin, marketed as Xenical^13^ and of Spiket-P, a potent cytotoxic drug against cancer cells.^14^ The product of the EH reaction with BGE *i.e.*, diol(*S*)-benzyloxy-1,2-propanediol also has significant applications. It is used in the synthesis of lipophilic aldehydes to inhibit human gastric lipase (HGL) and pancreatic lipase (HPL) and function as potent anti-obesity drugs.^15^ Additionally, it is used in the synthesis of the macrolide core of migrastatin, an anti-cancer drug.^16^ Thus, both (*R*)-BGE and the (*S*)-diol product have significant pharmaceutical relevance. Though resolution of BGE by EH from microorganisms such as *Rhodotorula mucilaginosa* and *Rhodococcus fascians* M022 have been reported, the enantioselectivity is low (3–7 *E*-value)^17,18^ and very few reports of EH with high enantioselectivity towards the pharmaceutically relevant BGE exist. In recent times attention has been focused on obtaining high enantioselectivity with high yields, the basis for enantioselectivity or the kinetics associated with the resolution of racemic epoxides has been less explored.

*Yarrowia lipolytica* NCIM 3589, isolated from oil contaminated areas of Mumbai High has been studied for its use in biodiesel production, biodegradation of brominated alkanes and synthesis of nanoparticles.^19–21^ A protein sequence with NCBI accession number XP_504164 (named as Ylehd) was cloned, expressed and purified as a 43 kDa protein.^22^ Ylehd, a bifunctional protein, shows epoxide hydrolase activity at pH 8.0 towards aliphatic and aromatic epoxides and promiscuous haloalkane dehalogenase activity at pH 4.5 towards halogenated compounds. Preliminary studies suggested that Ylehd was also able to hydrolyse PGE and BGE.^22^ In this study, we attempt to understand the basis for enantioselective properties of the protein by *in silico* techniques and *in vitro* assays wherein the (*S*)-selective resolution of BGE to (*S*)-3-benzyloxy-1,2-propanediol has been demonstrated with accumulation of (*R*)-BGE. Using steady-state kinetic analysis, thermodynamic studies and molecular dynamic simulations, the probable reason for the enantioselective behaviour of Ylehd has been suggested.

## Results and discussion

Specific activity of Ylehd with racemic PGE and BGE was found to be 18.23 and 25 U mg^−1^ protein, respectively. No activity could be detected with styrene oxide (SO) and 2-methyl-3-phenyloxirane (MPO) as substrates under the given assay conditions.

### Steady state kinetics of enantioselective hydrolysis by Ylehd

Studies with racemic (rac), (*R*) and (*S*) enantiomers of PGE and BGE with Ylehd suggest a substrate saturation profile as per the Michaelis Menten kinetics (Fig. S1†). Kinetic constants indicate (rac) BGE as a more suitable substrate for Ylehd. Though the turnover (*k*_cat_) of PGE (7.34 s^−1^) was higher than that of BGE (5.73 s^−1^), the specificity constant (*k*_cat_/*K*_m_) was greater for BGE (38.2 mM^−1^ s^−1^) than PGE (12.02 mM^−1^ s^−1^).

As seen in Table 1, catalytic efficiency (*k*_cat_/*K*_m_) and *K*_m_ values were found to be marginally higher for (*R*)-PGE (25.5 mM^−1^ s^−1^ and 1.32 mM) as compared to (*S*)-PGE (16.6 mM^−1^ s^−1^ and 0.93 mM) with a calculated *E*-value of 1.5 at 30 °C in 30 min. This was slightly lower than the *E* value of 4.6 for *Aspergillus niger* EH which showed slight selectivity in favour of (*S*)-PGE. Higher *E* value of 65 has been observed for TpEH1 from *Tsukamurella paurometabola* giving (*R*)-PGE with enantiomeric excess of more than 99% ee and 45% yield. BMEH from *Bacillus megaterium* ECU1001 preferentially hydrolysed the (*R*)-PGE yielding (*S*)-epoxide and (*R*)-diol with high enantioselectivity (*E* = 47.8) and enantiomeric excess of >99.5% and 55.9% yield.

**Table 1:** Kinetic parameters for Ylehd under steady-state conditions^a^

Compounds	Binding energy from docking studies (kJ mol^−1^)	*k*_303 K_ (μM^−1^ s^−1^)	*K*_m_ (mM)	*k*_cat_ b (s^−1^)	*k*_cat_/*K*_m_ (mM^−1^ s^−1^)	*E* c
rac-PGE	−25.73	17.3 + 0.82	0.6 ± 0.009	7.34 ± 0.06	12.2 ± 0.78	1.5
(*R*)-PGE	−26.27	28.11 + 0.73	1.32 ± 0.045	33.79 ± 0.87	25.5 ± 0.97	
(*S*)-PGE	−25.77	21.9 + 1.05	0.93 ± 0.071	15.66 ± 0.49	16.6 ± 0.057	
rac-BGE	−26.36	21.3 + 0.08	0.15 ± 0.002	5.73 ± 0.54	38.2 ± 1.08	10.37
(*R*)-BGE	−27.15	18.2 + 0.47	0.8 ± 0.01	3.45 ± 0.089	4.31 ± 0.07	
(*S*)-BGE	−28.41	31.1 + 0.97	0.27 ± 0.024	12 ± 0.97	44.7 ± 0.87	

a Kinetic constants calculated by non-linear regression analysis in Microcal Origin.

b Kinetic studies for all compounds were carried out with 2 μg protein except for (*R*)-BGE wherein 10 μg protein was used.

c Enantiomeric ratio (*E*) was calculated as ratio of *k*_cat_/*K*_m_ of the fast-acting enantiomer over the slow acting one.

Amongst the enantiomers, (*S*)-BGE seems to be the most suitable ligand for Ylehd both in terms of binding and catalysis. The *K*_m_ values for (*S*)-BGE (0.27 mM) were lower than that of (*R*)-BGE (0.8 mM). The *k*_cat_/*K*_m_ for (*S*)-BGE (44.7 mM^−1^ s^−1^) was greater than that for the (*R*)-enantiomer (4.31 mM^−1^ s^−1^), resulting in an *E* value of 10.37, calculated as a ratio of (*k*_cat_/*K*_m_)_S_ to (k_cat_/*K*_m_)_R_ at 30 °C. As seen in Table 1, Ylehd had a preference for (*S*)-BGE and the *E*-value for BGE was ∼7 fold higher than that for PGE. It is to be noted that though reports on enantioselective conversion of PGE exist, little information is available on the enantioselective conversion of BGE.

### Ylehd hydrolysis of racemic BGE determined by chiral HPLC

In order to validate the enantioselectivity of Ylehd for BGE, wherein the enzyme hydrolyses the racemic BGE converting the (*S*) form to the diol while retaining the (*R*)-enantiomer in the epoxide form, chiral HPLC analysis of the reaction mix was carried out. Separation of the two enantiomers was seen with retention times of 8.3 and 9.3 minutes for (*S*)-BGE and (*R*)-BGE, respectively (Fig. S2†). At the end of the reaction in a short time period of 20 minutes, the (*R*)-enantiomer was retained in the reaction mixture with 95% ee and yield of 10%. The diol formed in the reaction was seen to retain configuration since (*S*)-3-benzyloxy-1,2-propanediol was formed in the reaction while (*R*)-BGE was retained as analysed by chiral HPLC. This confirmed the (*S*)-selective nature of Ylehd for BGE.

It is to be noted that hydrolysis of the racemic forms would be majorly influenced by the differential behaviour of the individual enantiomers in the active site pocket of Ylehd. Thus, to study the mode of binding of the enantiomers in the active site pocket required modelling of Ylehd. As no crystal structure of the protein is available we attempted to model this bifunctional protein.

### Modelling of Ylehd and docking

Due to high diversity in sequence space and the availability of a relatively small number of crystal structures of EHs, the template showing best homology to Ylehd protein was identified as Murine EH (PDB ID: 1CQZ) with 29% sequence identity and 92% query coverage. Considering the low sequence similarity of Ylehd with 1CQZ, the second best template with 28% sequence identity and 40% query coverage from *Xanthobacter autotrophicus* exhibiting haloalkane dehalogenase activity (PDB: 1BEE) was additionally considered. Also to be noted that our earlier studies have shown Ylehd to be a bifunctional enzyme exhibiting epoxide hydrolase activity with promiscuous haloalkane dehalogenase (HLD) activity.^22^ Hence, the 2-template approach was adopted for building the structure of Ylehd to obtain the model. Both these templates belong to the α/β hydrolase superfamily and selection of these two templates covers more than 98% of the query sequence to be modelled. In another study, the structure of EH from *Mugil cephalus* has been built with considerable success by homology modelling using two templates, namely, EH from *A. niger* (1QO7) and *A. radiobacter* (PDB: 1EHY).^23^ Structure prediction for EH from *Agromyces mediolanus* has also been done using homology modelling wherein EH from *Streptomyces carzinostaticus* (PDB: 4I19) was considered as template.^24^

**Fig. 2:**
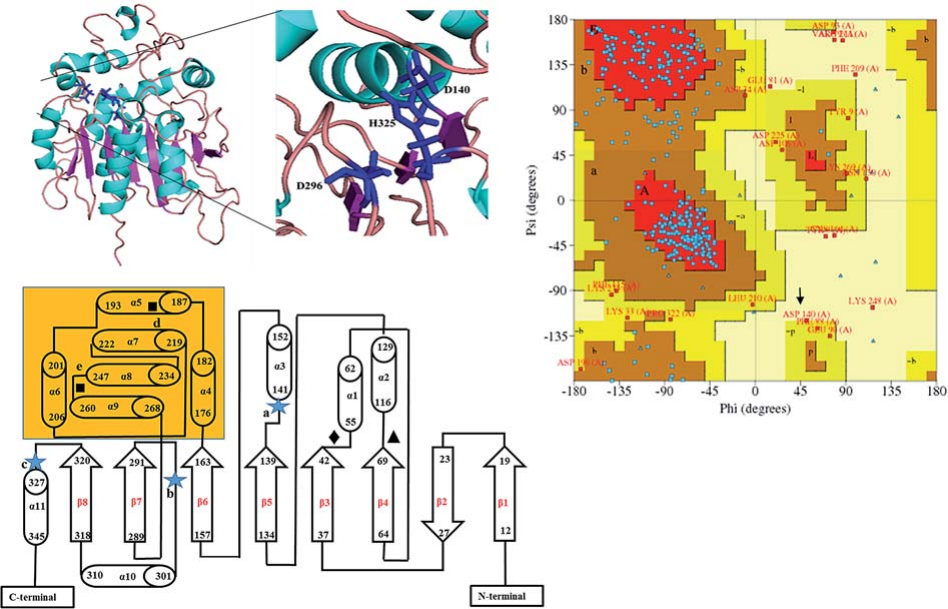
Structure of Ylehd (a) model representing alpha helices (cyan) and beta sheets (magenta) catalytic triad residues as indicated in the enlarged inset. (b) Protein topology showing shaded region as cap domain. Residues marked 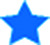 are catalytic triad residues-D140 (a), D296 (b), H325 (c), tyrosine residues marked with ■ Y189 (d), Y258 (e), HGFP motif ♦, GFPGS motif ▲ (c) Ramachandran plot distribution A – core alpha, L – core left-handed alpha, a – allowed alpha, l – allowed left-handed alpha, -a – generous alpha, -l – generous left-handed alpha, B – core beta, p – allowed epsilon, b – allowed beta, -p – generous epsilon, -b – generous beta. Red area corresponds to the ‘core’ regions representing the most favourable combinations of phi (*ϕ*)–psi (*ψ*) values. Brown corresponds to residues that are comparatively less favourable with respect to the allowed regions and generously allowed regions. Yellow corresponds to those residues seen in the ‘disallowed regions’ D140 indicated with arrow.

**Fig. 3:**
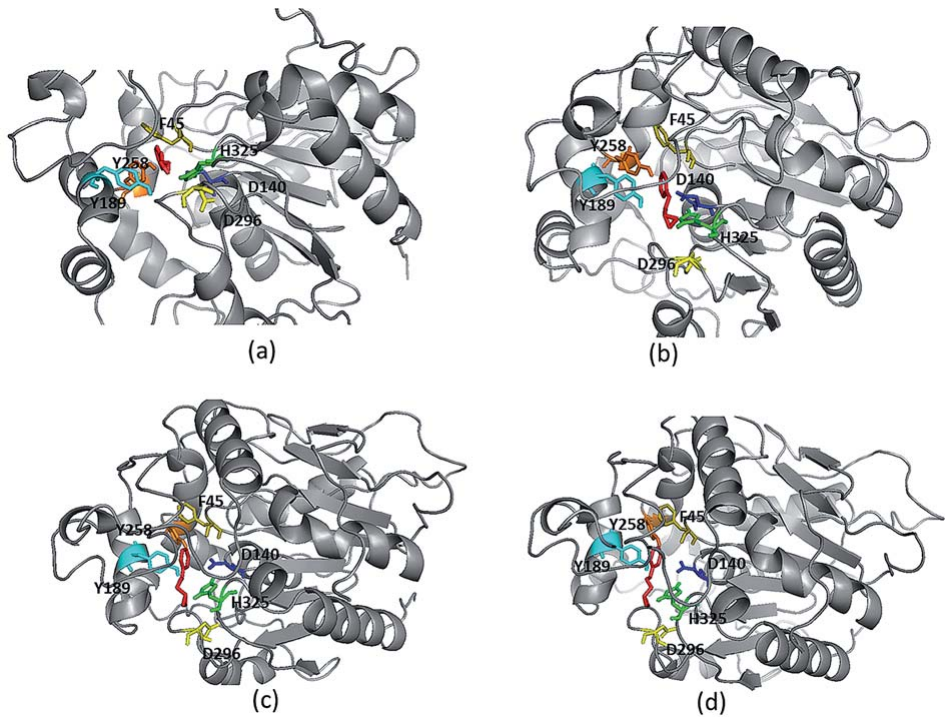
Ligand protein interaction for enantiomers (a) (*R*)-PGE, (b) (*S*)-PGE, (c) (*R*)-BGE, (d) (*S*)-BGE.

Models (20 numbers) were generated using MODELLER and ranked based on their energy value, *Z*-score and Ramachandran plot distribution (Table 2). The best model (number 3) with energy of −55 379.8 kJ mol^−1^ and *Z* score of −5.51 was selected for further studies. This model had a typical α/β hydrolase fold with a cap region exclusively consisting of α helices and a catalytic domain consisting of 7 parallel and one anti-parallel β-sheet (Fig. 2a). The topology of Ylehd (Fig. 2b) is similar to that of the general α/β-hydrolase fold EH proteins.^25^ The residues forming the cap region with the conserved tyrosine residues are seen to consist of only α helices as seen for soluble EH proteins while the catalytic domain consists of parallel and anti-parallel β sheets followed by loops bearing the predicted triad residues, namely, D140, D296 and H325. The nucleophile elbow, formed by G-F-P-G-S in Ylehd present on a loop between strand β5 and the following helix α3 contained the nucleophile D140 as is true for most EH proteins.^26^ To further assess the quality of the model, analysis using Ramachandran plot was also carried out (Fig. 2c). Number of residues reported to be in the most favoured regions, in the additionally allowed regions, in generously allowed regions were 84, 11.3 and 2.3%, respectively, while 2% of the residues were in the disallowed regions of the Ramachandran plot. It has been shown that α/β hydrolase proteins have a characteristic Ramachandran plot wherein the nucleophile of the catalytic triad falls in the disallowed region.^26^ This was true even for Ylehd wherein D140, assumed to be the nucleophile, also falls in the disallowed region. Till date crystal structures of EH from bacterial, fungal, plant and mammalian sources reported in the PDB database adopt a canonical α/β-hydrolase fold with cap region and a catalytic domain and similar features have been observed in the model for Ylehd. Further, to investigate the substrate selectivity of Ylehd, the energy minimized model was used and 9 aliphatic and 4 aromatic epoxides (Fig. S3†) were docked flexibly and their binding energies compared. The aromatic glycidyl ethers, rac PGE and BGE interacted at the active site with binding energy of −25.73 and −26.35 kJ mol^−1^, respectively. It is to be noted that while *k*_cat_ values for PGE were much higher than those seen for BGE, Ylehd was found to have a greater affinity towards (*S*)-BGE as inferred from its *K*_m_ value. Enantiomers of PGE and BGE were found to bind to the same active site with differences seen in the orientation of the ligands. As seen in Table 1, binding energies of −26.27 and −25.77 kJ mol^−1^ were obtained for (*R*)- and (*S*)-PGE, respectively while lower values of −27.15 and −28.41 kJ mol^−1^ were obtained for (*R*)- and (*S*)-BGE, respectively. All the four poses show the presence of the catalytic triad residues (D140, D296, H325), the tyrosines (Y189, Y258) and the key residue of the oxyanion hole (F45) as seen in Fig. 3.

**Table 2:** Comparative scores of generated models

Model number	Energy values (kJ mol^−1^)	*Z*-score from Pros-A	Ramachandran plot distribution^a^
3	−55 379.8	−5.51	84, 11.3, 2.7, 2.0
8	−55 494.4	−5.02	81.3, 13, 3.7, 2.0
11	−54 134.3	−5.35	84, 11.3, 2.7, 2.0
12	−55 411.8	−4.78	77.7, 15.7, 4.7, 2.0
17	−54 140.3	−4.88	79.3, 15.3, 3.3, 2.0

a Values in the Ramachandran plot distribution are the percentage of residues in the four quadrants of the plot.

### Thermodynamic parameters of Ylehd

**Fig. 4:**
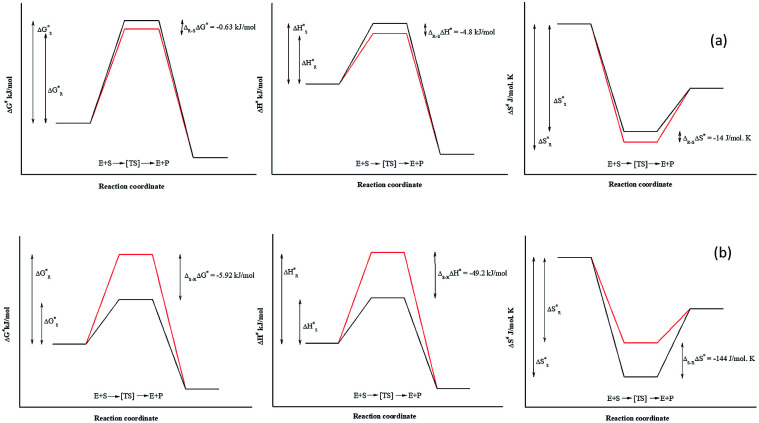
Schematic diagram of thermodynamic determinants for enantioselective property of Ylehd reaction coordinates for Ylehd catalysed resolution of PGE (a) and BGE (b) with enantiomers designated as *R* (red) and *S* (black). Δ*G*^#^, Δ*H*^#^, Δ*S*^#^ values calculated by subtracting Δ*G*^#^, Δ*H*^#^, Δ*S*^#^ values of the fast acting (*R*)-PGE enantiomer from that of the slower (*S*)-PGE enantiomer. ΔΔ*G*^#^, ΔΔ*H*^#^, ΔΔ*S*^#^ values calculated by subtracting Δ*G*^#^, Δ*H*^#^, Δ*S*^#^ values of the fast acting (*S*)-BGE enantiomer from that of the slower (*R*)-BGE enantiomer.

Due to the identical ground state energies of the enantiomers, enzyme enantioselectivity can be reduced to the comparison of difference in the energetics of transition state of the enantiomers. Thus, enantioselectivity can be caused by difference in activation free energy between the enantiomers and can be separated into enthalpic and entropic terms of activation. The Arrhenius energy of activation (*E*_a_) was determined for Ylehd with respect to PGE and BGE (Table 3) and found to be marginally higher for (*S*)-PGE at 72 kJ mol^−1^ as compared to (*R*)-PGE (67.5 kJ mol^−1^) with a difference of 4.8 kJ mol^−1^ between the two enantiomers at 30 °C. In contrast, *E*_a_ was significantly higher for (*R*)-BGE (75.2 kJ mol^−1^) than that for (*S*)-BGE (26 kJ mol^−1^) with a difference of 49.2 kJ mol^−1^ suggesting that this difference in the activation energy favours (*S*)-BGE as the substrate for EH activity of Ylehd at 30 °C. The estimated difference in Gibbs free energy of activation (Δ*G*^#^) for all compounds was <0 suggesting that all the reactions were spontaneous and thermodynamically favourable. The Δ*G*^#^ value was found to be lowest for (*S*)-BGE (−5.69 kJ mol^−1^) as compared to the other enantiomers. From the temperature dependence of *E*, values for activation enthalpy (Δ*H*^#^) and entropic term (*T*Δ*S*^#^) were ascertained and were found to be suitable for (*S*)-BGE (Table 3). Enantioselectivity arises due to the difference in the free energy of activation between the enantiomers with contributions from activation enthalpic and entropicterms. For BGE, the experimentally determined differential activation free energy (ΔΔ*G*^#^) values for the individual enantiomers was found to be −5.43 kJ mol^−1^ while the calculated ΔΔ*G*^#^ values from eqn (2) were found to be −6.04 kJ mol^−1^. Thus, the calculated and experimental values were seen to corroborate with each other. Similar correlations were also studied for PGE wherein the ΔΔ*G*^#^ values from Δ*G*^#^ values of individual enantiomers were found to be −0.63 kJ mol^−1^ and calculated value from eqn (2) was found to be −0.79 kJ mol^−1^. It can be seen from Fig. 4 that ligands that show greater ΔΔ*G*^#^, ΔΔ*H*^#^ and ΔΔ*S*^#^ between their specific enantiomers, are more favourable substrates for enantioselective resolutions. ΔΔ*H*^#^ and ΔΔ*S*^#^ values are positive which indicated that the reaction was spontaneous at high temperatures. As suggested by Ottosson *et al.* (2002), a better transition state binding (low enthalpy) comes with a more rigid transition state (low entropy) *i.e.*, an enantiomer favoured by enthalpy is disfavoured by entropy.^27^ This seems to be the case with (*S*)-BGE, the enantiomer best acted upon by Ylehd. Since temperature was seen to play a role on the efficiency of the reaction it was noteworthy to find the racemic temperature *T*_R_ wherein both enantiomers are expected to be present in equal amounts. The temperature for PGE was fond to be 300 K (27 °C). The *E* value at this temperature was expected to be 1.0. As per the kinetic studies carried out at 30 °C, *E* value was fund to be 1.5. Hence it can be clearly seen that the thermodynamic and kinetic studies seem to correlate well. For BGE, the *T*_R_ was found to be 342 K (69 °C) which could be the temperature at which the *E* value would be 1.0. Thus, the enantioselectivity exhibited by Ylehd was temperature dependent which varied between compounds and could be a parameter to consider while optimizing the process for resolution of racemic mixtures with high enantioselectivity and better yields.

**Table 3:** Thermodynamic parameters for activation that determine the kinetic resolution of rac-PGE and rac-BGE catalysed by Ylehd^a^

Name of compound	*E*_a_ (kJ mol^−1^)	Δ*G*^#^ (kJ mol^−1^), 303K	Δ*H*^#^ (kJ mol^−1^), 303K	*T*Δ*S*^#^ (kJ mol^−1^), 303K	Δ*S*^#^ (J mol^−1^ K^−1^), 303K	*T*_R_ (K)
(*R*)-PGE	67.5 ± 2.3	−1.32 ± 0.2	65 ± 3.1	66.32 ± 2.47	219 ± 1.05	300
(*S*)-PGE	72.3 ± 3.8	−0.69 ± 0.04	69.8 ± 2.04	70.49 ± 3.09	233 ± 1.2	
(*R*)-BGE	75.2 ± 2.9	−0.23 ± 0.05	72.68 ± 1.98	72.90 ± 2.01	240 ± 1.3	342
(*S*)-BGE	26 ± 1.6	−5.69 ± 0.29	23.48 ± 2.13	29.17 ± 1.48	96 ± 1.9	

a All values were calculated as average of 3 sets of experimental replicates and were considered statistically significant with p < 0.005. Arrhenius energy (E_a_) of activation was calculated by plotting the ln k where k is rate constant at each temperature against reciprocal of temperature (1/T) in kelvin.

### Molecular dynamics simulation of Ylehd

**Fig. 5:**
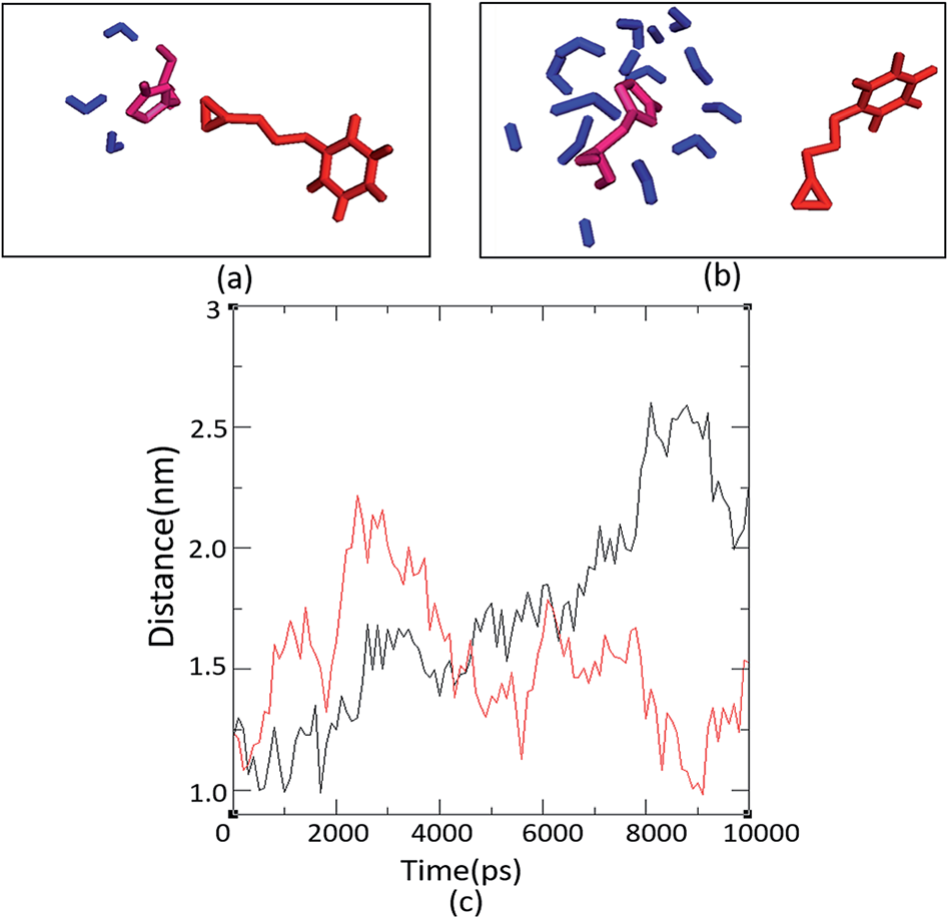
Hydrated active site pocket of Ylehd bound with (*R*)-BGE and (*S*)-BGE and distance analysis. Water molecules seen around H325 with (*R*)-BGE (a) and (*S*)-BGE (b) bound to Ylehd after MD simulation (10 ns). Trajectory of distance between H325 of Ylehd (*R*)-BGE (red) and (*S*)-BGE (black) bound form and water throughout the simulation of 10 ns (c). The distance is an average of all the water molecules present at the active site pocket of Ylehd bound individually to the two ligands.

The number and proximity of water molecules near the base H325 at the active site, an important residue in the charge relay, were studied by molecular dynamic simulations for Ylehd complexed with (*S*)-BGE and (*R*)-BGE. The number of water molecules (∼11 numbers) are more around (*S*)-BGE bound to Ylehd (Fig. 5b) than around (*R*)-BGE (∼3 numbers) (Fig. 5a). Furthermore, up to 4 ns the base H325 of Ylehd bound to (*S*)-BGE was closer to the water molecules than that for (*R*)-BGE with a distance as low as 1 Å (Fig. 5c). This difference between the distances may have a role in conversion of (*S*)-BGE by faster activation of water molecules by the likely charge relay system of D296 and His 325 leading to hydrolysis of acyl-enzyme intermediate complex and release of the diol.

Earlier studies on mutants of *Aspergillus niger* generated by directed evolution, have shown that the distance (*d*) between nucleophile and carbon C1 (*d*1) or carbon C2 (*d*2) of the bound epoxide oxirane ring may suggest propensity of attack on the ring carbons which in turn could determine the enantioselective property or *E* value of the enzyme. Attack at the C1 would lead to the (*R*)-diol product while that at C2 would form the (*S*)-diol product.^28,29^ In this study, docking after energy minimization showed that in Ylehd bound to (*S*)-BGE, *d*1 and *d*2 were 4.5 and 5.7 Å, respectively while in the case of (*R*)-BGE bound docked pose, *d*1 and *d*2 are 6.9 and 8.3 Å, respectively (Fig. S4†). Further, the distance trajectories of these bound forms in the hydrated active site pocket after molecular docking simulations of the docked poses were also analysed. In case of (*S*)-BGE *d*1 > *d*2 up to 4 ns which would facilitate the preference of the (*S*)-enantiomer and also formation of the (*S*)-diol product as has been seen in the *in vitro* assays. However, in case of (*R*)-BGE, though *d*1 and *d*2 are less than that in (*S*)-BGE (Fig. S4†), (*R*)-BGE still remains the less preferred substrate. This could be arising either due to high propensity of steric clashes between the interacting atoms or due to the presence of lower number of water molecules and greater distance between water and H325 in the (*R*)-BGE bound Ylehd. Thus, this study further emphasizes the role of water and the propensities of water to H325 as key contributors to enantioselective property of Ylehd.

BGE is an attractive intermediate in synthetic reactions since the bulkiness of the benzyl group makes it an easy target for deprotection.^30^ Both (*R*)-BGE and the (*S*)-diol product have significant pharmaceutical relevance. A few studies on resolution of BGE with variety of microbial strains have been reported in literature (Table 4). It is to be noted that most of these studies have been carried out using whole cells. Bioconversions with whole cells need incubation times from 25–120 h since biomass generation is the rate limiting step in the process. Also, mass transfer of the relatively hydrophobic aryl glycidyl compound and product release in whole cell biocatalysis by intracellular enzymes or stability of extracellular enzymes, are major factors contributing to the efficiency of the process. Use of purified enzymes can eliminate the problems of by-products formed by side reactions, leading to higher purity and easier downstream processing.

**Table 4:** Strains used for the kinetic resolution of (rac)-BGE

Name of the organism	Type of biocatalyst	*E* value	%ee	Remarks
*Talaromyces flavus* ^31^	Whole cells	13 (*R*)	96	Whole cell biotransformation after growing cells for 72 h and reaction time of 4 h
*Rhodococcus fascians* M022	Whole cells	3–4 (*S*)	45	Biotransformation completion occurs in 12–24 h
*Rhodocccus mucilaginosa* M002 (ref. 17)	Whole cells	7–8 (*R*)	77	
*Aspergillus sydowii* ^32^	Whole cells	*E*-value not specified (*R*)	46	Maximum % ee obtained after growing cells for 120 h and 120 h of biotransformation
*Trichoderma* sp.^32^	Whole cells	*E*-value not specified (*S*)	60	Maximum % ee obtained after growing cells for 120 h of and growth and after 25 h of biotransformation
*Agromyces mediolanus* ZJB120203 (ref. 24)	Purified protein	*E*-value not specified (*S*)	>99	Resolution of BGE has been done in this study but epichlorohydrin remains the most suitable substrate for this protein
*Yarrowia lipolytica* NCIM 3589 (this study)	Purified protein (Ylehd)	10.37 (*R*)	95	Catalysis brought about by purified enzyme in 30 min

## Experimental

### Chemicals

Medium components and supplements were obtained from HiMedia Laboratories Pvt. Ltd (Mumbai, India). Solvents and compounds were procured from Sisco Research Laboratories Pvt. Ltd (Mumbai, India), Merck Millipore Company (Mumbai, India), Sigma Aldrich (Missouri, USA) and, racemic, *R* and *S* forms of phenyl and benzyl glycidyl ether were obtained from TCI chemicals (Tokyo, Japan). All chemicals used in the study were >99% pure as per the manufacture's specifications. Molecular biology reagents and kits were sourced from Bangalore Genei Chemicals (Bangalore, India).

### Strains and media

*Yarrowia lipolytica* NCIM 3589 cells was grown for 24 h on Yeast extract Peptone Dextrose (YPD) medium containing (g L^−1^): yeast extract, 10.0; peptone, 20.0; dextrose, 20.0. Genomic DNA extraction was done according to Sambrook and Russell (2001). *Escherichia coli* DH5α and *Escherichia coli* BL21AI obtained from Invitrogen, (California, USA) were used for clone construction and expression studies, respectively. Both strains of *E. coli* were routinely grown and maintained on Luria Bertani (LB) broth or agar supplemented with 50 μg ml^−1^ Kanamycin when necessary.

### Spectrophotometric assay for determination of epoxide hydrolase activity

EH activity was determined spectrophotometrically at 590 nm by measuring the residual epoxide glycidyl ethers.^32^ For determination of kinetic constants varying concentrations of the substrate were added to the reaction mixture which contained, 2 μg of pure protein in 50 mM Tris pH 8.0, kept at 30 °C for 30 minutes. One unit of enzyme activity was defined as the amount of enzyme required for conversion 1 micromole of substrate to product per minute under the given assay conditions.

### Enzyme activity, steady-state kinetics and thermodynamics

The protein was cloned and expressed in *E. coli* BL21AI strain. EH activity was determined spectrophotometrically at 590 nm by measuring the residual epoxide glycidyl ethers.^33^

For determination of kinetic constants, the reaction mixture containing varying concentrations of the substrate in 50 mM Tris buffer, pH 8.0, 2 μg of pure protein and were incubated at 30 °C for 30 minutes. One unit of enzyme activity was defined as the amount of enzyme required for conversion 1.0 micromole of substrate to product per minute under the given assay conditions.

Kinetic constants, *K*_m_, *V*_max,_ turnover numbers (*k*_cat_) and the catalytic efficiency (*k*_cat_/*K*_m_), were calculated from the initial velocity measurements by Michaelis–Menten equation using non-linear curve fitting in Origin ver 8.5 (Microcal Ltd, U.S.A). For determination of enantiomeric ratio (*E*) of the enzyme varying concentrations of pure enantiomers of glycidyl ethers, were used and calculated as a ratio of the *k*_cat_/*K*_m_ of the more reactive to the less reactive enantiomer. Energy of activation (*E*_a_) was calculated for the enantiomers by plotting a graph of ln *k versus* 1/*T* where *k* is the rate constant at each temperature and *T* is the temperature in kelvin (K). Activation enthalpy (Δ*H*^#^), entropy (Δ*S*^#^) Gibb's free energy of activation (Δ*G*^#^) and racemic temperature (*T*_R_) were calculated as per the conventional thermodynamic equations as shown below:^34^

1 Δ*G*^#^ = −*RT* ln *k*,

where *R* = universal gas constant and *k* is the rate constant at 303 K,

2 ΔΔ*G*^#^ = −*RT* ln *E*

For each enantiomer,

3 Δ*H*^#^ = *E*_a_ − *RT*

4 *T*Δ*S*^#^ = Δ*H*^#^ − Δ*G*^#^

### Resolution of racemic BGE and detection of diol

Racemic benzyl glycidyl ether was added to 50 mM Tris pH 8.0 at a concentration of 20 mM in ethanol and reaction was initiated by addition of 36 μg of purified enzyme, kept at 30 °C up to 40 minutes in shaking water bath. At the end of the incubation time the reaction mix was extracted 1 : 1 with 95 : 5 hexane: isopropanol. The organic layer was injected on a Chiralpak IC column (250 × 4.6 μm, Daicel, Tokyo, Japan) in a isocratic mode with the mobile phase 95 : 5 hexane : isopropanol at room temperature with a flow rate of 0.5 ml min^−1^ at 240 nm on Shimadzu UFLC 6-AD. In order to detect the diol product in the reaction, reaction mixture was lyophilized completely, re-dissolved in isopropanol and then injected on Chiralpak IG-3 column (250 × 4.6 μm, Daicel, Tokyo, Japan) in a isocratic mode with the mobile phase 90 : 05 : 05 *n*-hexane : ethanol : methanol at 15 °C with a flow rate of 1 ml min^−1^ at 220 nm on Shimadzu UFLC 6-AD.

### Homology modelling of Ylehd

The PDB database was searched using BLAST-P^35^ for suitable template to be used in homology modelling. Two templates were selected based on identity and query coverage. Multiple sequence alignment was generated using CLUSTALW.^36^ The secondary structural features for the query were predicted using PSIPRED tool.^37^ JOY was used to map the secondary structures for the template and annotations were then incorporated in the alignment between query and the templates.^38^ The alignment was used to generate the 3-D coordinates of the query using MODELLER.^39^ The models were validated based on their Ramachandran plot distribution and *Z*-score.^40,41^ The best model was energy minimized using the SYBYL 7.2 software (Tripos Inc. Ltd), Tripos force field and Powell's gradient until convergence. The energy minimized model was considered for further loop modelling (wherever templates were unavailable for loops) in SYBYL 7.2 software. The final structure was then energy minimized using the same parameters successively after construction of every loop region. Structures were viewed and analysed using PyMOL (The PyMOL Molecular Graphics System, Version 1.8 Schrödinger, LLC).

### Computational enzyme–substrate docking analysis

Computational ligand docking studies were carried out using Autodock Version 4.2.6.^42^ The homology model of Ylehd protein obtained in the previous step was used as the receptor and each of the substrates was used as the ligand. 3D coordinates of the ligands were downloaded from Pubchem and the ids are as mentioned in Fig. S3.† Both the protein and ligand were pre-processed (addition of hydrogens and calculation of charges for protein and assigning torsions to the ligands) as per the Autodock protocol. The grid was set for the full protein of size 120 × 120 × 120 and a spacing of 0.375 Å. Coordinates of Central Grid Point of Maps were 43.915, 43.992 and 43.925. Docking was carried out with flexible ligand for 100 genetic algorithm runs with a population size of 100 and a maximum of 27 000 generations. The interactions in the protein–ligand complexes were analysed using PyMOL and Ligplot.^43^

### Molecular dynamic simulation

Molecular dynamics simulations were carried out for the protein–ligand complexes to observe the presence of water molecules at the binding site as water is known to play an important role in catalysis. MD simulations were carried out for Ylehd protein in complex with (*S*)-BGE and (*R*)-BGE (ligands) for 10 ns each. The GROMACS molecular modelling software (version 5.5) was employed for performing the MD simulations of the protein–ligand complexes. Topology files for the protein and the ligand were created using pdb2gmx. The topology file for the small molecule ligand was generated using the PRODRG server.^44^ A dodecahedron box was utilized for the protein–ligand complex. The complexes were placed in their respective boxes such that the distance between the complex and the edge of the box was at least 10 Å and solvated using the SPC216 water model. Nineteen sodium ions were added at random positions into the solvent to neutralize the system. Energy minimization to relax internal constraints of the complexes was performed using the method of steepest descent, with no position restraints applied. Following this, the complexes were subjected to 300 ps of equilibration to heat the system to 300 K with position restraints applied. Subsequently, another round of equilibration under normal pressure temperature conditions was performed for 200 ps to bring the atmospheric pressure of the system to 1 bar. A time step of 1 femtosecond was used for both the equilibration runs. After this, 100 ns of production MD under NPT conditions was initiated using the leap-frog integrator with a time step of 2 femtoseconds. V-rescale thermostat^45^ and Parrinello–Rahmanbarostat^46^ was used to maintain temperature and pressure respectively of the system. Coordinates were saved every 10 ps. Graphs were generated using the Xmgrace plotting tool.

## Conclusions

In this study, we report on the kinetic resolution of glycidyl ethers by Ylehd from *Yarrowia lipolytica* as an epoxide hydrolase belonging to the α/β hydrolase family and is the first report of an enantioselective EH from this yeast. Homology modelling using a two-template approach shows that the protein has a canonical α/β hydrolase fold. The enzyme showed enantioselectivity towards (*S*)-BGE hydrolysing it to its corresponding vicinal diol while retaining (*R*)-BGE up to 95% ee in a short time period of 20 min. The selectivity of Ylehd for (*S*)-BGE could be correlated to its kinetic and thermodynamic parameters as well as to the number and proximity of water molecules near the base H325 in the active site pocket. Thus, this study gives an insight into the role played by kinetic and thermodynamic determinants along with the function of water in the enantioselective conversion of benzyl glycidyl ether catalysed by Ylehd. The results obtained from this study will be used to engineer a robust biocatalyst and optimize the process parameters for conversion of glycidyl ethers with higher yields.

## Conflicts of interest

There are no conflicts to declare.

## Supplementary Material

RA-008-C8RA00628H-s001

## References

[cit1] Tokunaga M., Larrow J. F., Kakiuchi F., Jacobsen E. N. (1997). Science.

[cit2] Schaus S. E., Brandes B. D., Larrow J. F., Tokunaga M., Hansen K. B., Gould A. E., Furrow M. E., Jacobsen E. N. (2002). J. Am. Chem. Soc..

[cit3] de Vries E. J., Janssen D. B. (2003). Curr. Opin. Biotechnol..

[cit4] Gill S. S., Hammock B. D. (1981). Biochem. Pharmacol..

[cit5] Schiøtt B., Bruice T. C. (2002). J. Am. Chem. Soc..

[cit6] Morisseau C., Nellaiah H., Archelas A., Furstoss R., Baratti J. C. (1997). Enzyme Microb. Technol..

[cit7] Lee J. W., Lee E. J., Yoo S. S., Park S. H., Kim H. S., Lee E. Y. (2003). Biotechnol. Bioprocess Eng..

[cit8] Wu S., Li A., Chin Y. S., Li Z. (2013). ACS Catal..

[cit9] Botes A. L., Weijers C. A. G. M., Botes P. J., van Dyk M. S. (1999). Tetrahedron: Asymmetry.

[cit10] Kong X.-D., Ma Q., Zhou J., Zeng B.-B., Xu J.-H. (2014). Angew. Chem., Int. Ed..

[cit11] Wu K., Wang H., Sun H., Wei D. (2015). Appl. Microbiol. Biotechnol..

[cit12] Xu Y., Xu J.-H., Pan J., Tang Y.-F. (2004). Biotechnol. Lett..

[cit13] Yadav J. S., Reddy M. S., Prasad A. R. (2006). Tetrahedron Lett..

[cit14] Uckun F. M., Mao C., Vassilev A. O., Huang H., Jan S.-T. (2000). Bioorg. Med. Chem. Lett..

[cit15] Kotsovolou S., Verger R., Kokotos G. (2002). Org. Lett..

[cit16] Gaul C., Danishefsky S. J. (2002). Tetrahedron Lett..

[cit17] Kotik M., Brichac J., Kyslík P. (2005). J. Biotechnol..

[cit18] Ye Q. , Bao J. and Zhong J.-J., Bioreactor Engineering Research and Industrial Applications I: Cell Factories, Springer, 2016

[cit19] Katre G., Joshi C., Khot M., Zinjarde S., RaviKumar A. (2012). AMB Express.

[cit20] Vatsal A., Zinjarde S. S., Kumar A. R. (2015). Biodegradation.

[cit21] Agnihotri M., Joshi S., Kumar A. R., Zinjarde S., Kulkarni S. (2009). Mater. Lett..

[cit22] Bendigiri C., Zinjarde S., RaviKumar A. (2017). Sci. Rep..

[cit23] Choi S. H., Kim H. S., Lee E. Y. (2009). Biotechnol. Lett..

[cit24] Xue F., Liu Z.-Q., Zou S.-P., Wan N.-W., Zhu W.-Y., Zhu Q., Zheng Y.-G. (2014). Process Biochem..

[cit25] van Loo B., Kingma J., Arand M., Wubbolts M. G., Janssen D. B. (2006). Appl. Environ. Microbiol..

[cit26] Ollis D. L., Cheah E., Cygler M., Dijkstra B., Frolow F., Franken S. M., Harel M., Remington S. J., Silman I., Schrag J., others (1992). Protein Eng..

[cit27] Ottosson J., Fransson L., Hult K. (2002). Protein Sci..

[cit28] Kotik M., Archelas A., Faměrová V., Oubrechtová P., Křen V. (2011). J. Biotechnol..

[cit29] Reetz M. T., Bocola M., Wang L.-W., Sanchis J., Cronin A., Arand M., Zou J., Archelas A., Bottalla A.-L., Naworyta A., Mowbray S. L. (2009). J. Am. Chem. Soc..

[cit30] Kasai N., Suzuki T., Furukawa Y. (1998). J. Mol. Catal. B: Enzym..

[cit31] Wei C., Chen Y., Shen H., Wang S., Chen L., Zhu Q. (2012). Biotechnol. Lett..

[cit32] Martins M. P., Mouad A. M., Boschini L., RegaliSeleghim M. H., Sette L. D., Meleiro Porto A. L. (2011). Mar. Biotechnol..

[cit33] Kumar R., Wani S. I., Chauhan N. S., Sharma R., Sareen D. (2011). Protein Expression Purif..

[cit34] Phillips R. S. (1996). Trends Biotechnol..

[cit35] Madden T. L., Tatusov R. L., Zhang J. (1996). Methods Enzymol..

[cit36] Thompson J. D., Higgins D. G., Gibson T. J. (1994). Nucleic Acids Res..

[cit37] Buchan D. W. A., Minneci F., Nugent T. C. O., Bryson K., Jones D. T. (2013). Nucleic Acids Res..

[cit38] Mizuguchi K., Deane C. M., Blundell T. L., Johnson M. S., Overington J. P. (1998). Bioinformatics.

[cit39] Webb B. and Sali A., in Current Protocols in Bioinformatics, ed. A. Bateman, W. R. Pearson, L. D. Stein, G. D. Stormo and J. R. Yates, John Wiley & Sons, Inc., Hoboken, NJ, USA, 2014, pp. 5.6.1–5.6.32

[cit40] Laskowski R. A., MacArthur M. W., Moss D. S., Thornton J. M. (1993). J. Appl. Crystallogr..

[cit41] Wiederstein M., Sippl M. J. (2007). Nucleic Acids Res..

[cit42] Morris G. M., Huey R., Lindstrom W., Sanner M. F., Belew R. K., Goodsell D. S., Olson A. J. (2009). J. Comput. Chem..

[cit43] Wallace A. C., Laskowski R. A., Thornton J. M. (1995). Protein Eng., Des. Sel..

[cit44] Schüttelkopf A. W., van Aalten D. M. F. (2004). Acta Crystallogr., Sect. D: Biol. Crystallogr..

[cit45] Bussi G., Donadio D., Parrinello M. (2007). J. Chem. Phys..

[cit46] Parrinello M. (1981). J. Appl. Phys..

