# Supplementary material for: Evaluating Ylehd, a recombinant epoxide hydrolase from Yarrowia lipolytica as a potential biocatalyst for the resolution of benzyl glycidyl ether
Source: RSC Adv. 2018 Apr 6;8(23):12918–26. doi: 10.1039/c8ra00628h (PMC9079618; doi:10.1039/c8ra00628h)
Supplement: RA-008-C8RA00628H-s001 [file RA-008-C8RA00628H-s001.pdf]

Electronic Supplementary Material (ESI) for RSC Advances.
This journal is © The Royal Society of Chemistry 2018

Evaluating Ylehd, a recombinant epoxide hydrolase from *Yarrowia lipolytica*as a potential biocatalyst for resolution of benzyl glycidyl ether

Chandrika Bendigiri[a], K. Harini[c], Sajal Yenkar[a], Smita Zinjarde[a], R.Sowdhamini[c] and Ameeta RaviKumar[a,b] *

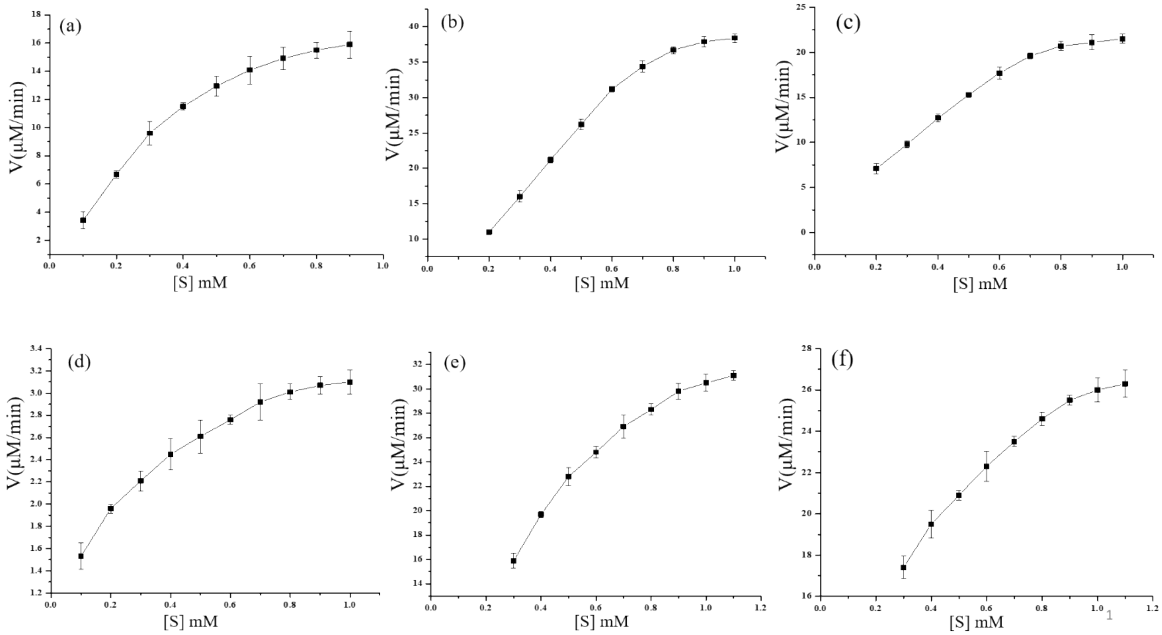

Fig. S1: Substrate saturation plots

Curves indicate the substrate saturation of Ylehd with rac-PGE(a), *(R)*-PGE(b), *(S)*-PGE(c), rac-BGE(d), *(R)*-BGE(e) and *(S)*-BGE(f)

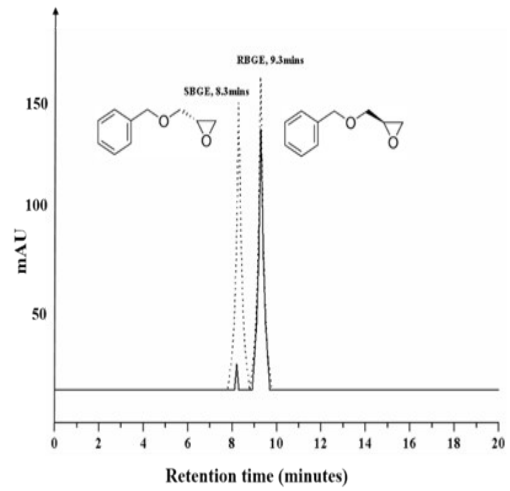

Figure S2: Resolution of BGE

Racemic BGE(dotted),Racemic BGE +Ylehd after 20 minutes of reaction(solid)

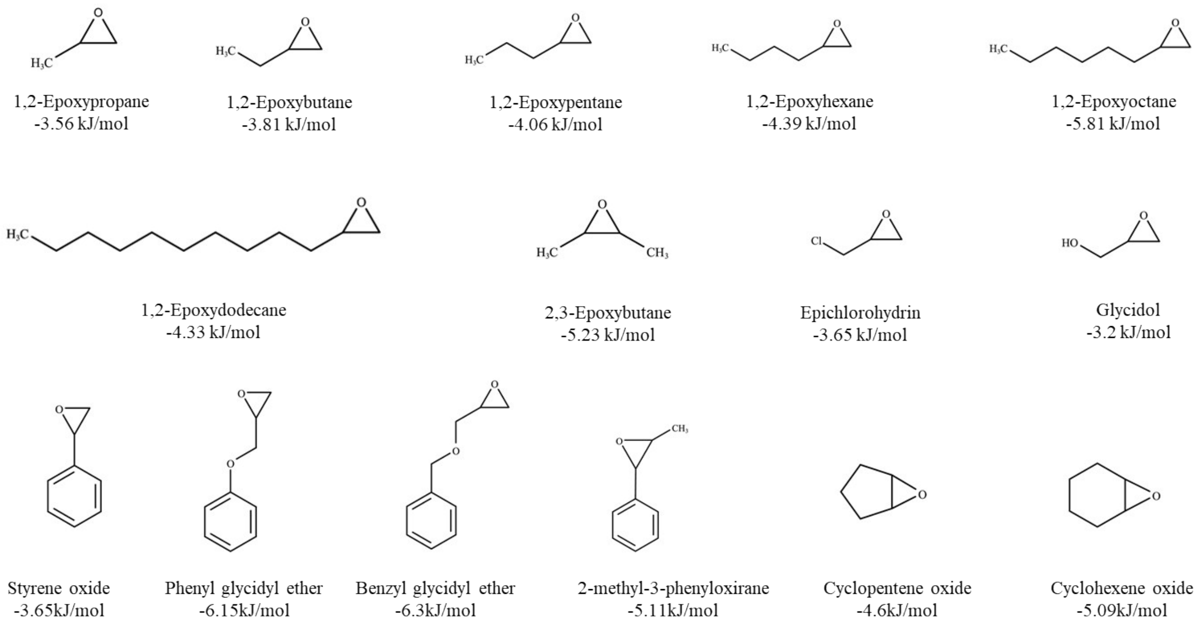

Fig. S3: Binding energy of compounds docked with Ylehd

3D coordinates of ligands were downloaded from Pubchem and docked flexibly with Ylehd protein model. Pubchem CID numbers are serially given as follows: 6378, 7834, 13853, 15036, 18126, 17858, 30664, 7835, 11164, 7276, 31217, 94247 and 12339.

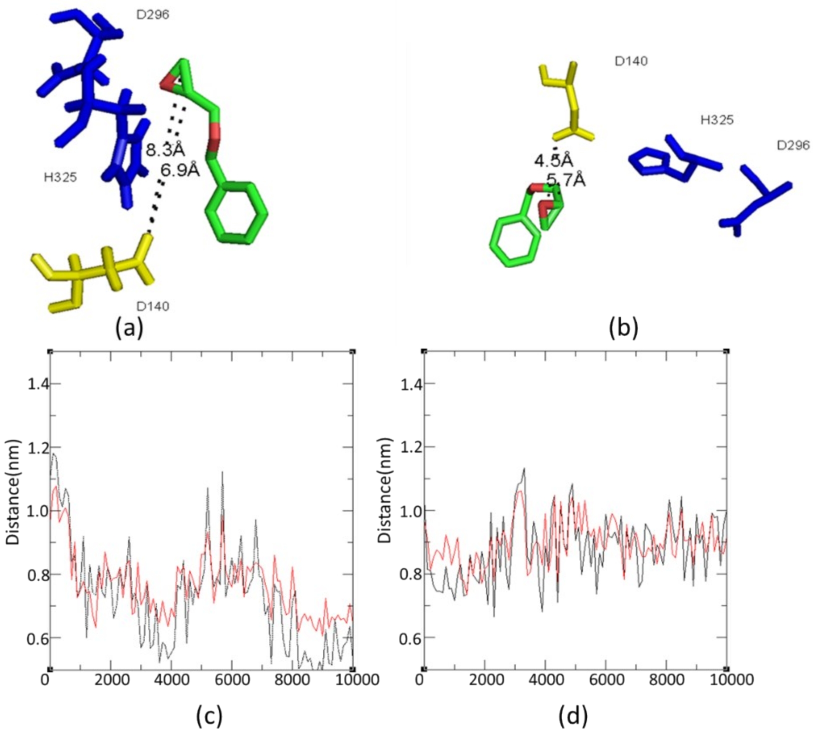

Fig. S4: Distance between nucleophile and ligand

(a,b) Distance between nucleophile D140 and carbon atoms of the oxirane ring of *(R)*-BGE(a) and (S)-BGE(b) bound to Ylehd model after docking and energy minimisation.

(c,d) Distance trajectories for C1(red) and C2(black) from D140 in hydrated active site pocket of Ylehd bound to *(R)*-BGE(c) and (S)-BGE(d) during the course of simulations.
